# Anti-Biofilm Activity of the Fungal Phytotoxin Sphaeropsidin A against Clinical Isolates of Antibiotic-Resistant Bacteria

**DOI:** 10.3390/toxins12070444

**Published:** 2020-07-08

**Authors:** Emanuela Roscetto, Marco Masi, Matilde Esposito, Roberta Di Lecce, Antonella Delicato, Lucia Maddau, Viola Calabrò, Antonio Evidente, Maria Rosaria Catania

**Affiliations:** 1 Dipartimento di Medicina Molecolare e Biotecnologie Mediche, Università di Napoli Federico II, Via Pansini 5, 80131 Naples, Italy; matilde.esposito87@gmail.com (M.E.); mariarosaria.catania@unina.it (M.R.C.); 2 Dipartimento di Scienze Chimiche, Università di Napoli Federico II, Complesso Universitario Monte S. Angelo, Via Cintia 4, 80126 Napoli, Italy; roberta.dilecce@unina.it (R.D.L.); evidente@unina.it (A.E.); 3 Dipartimento di Biologia, Università di Napoli Federico II, Complesso Universitario Monte S. Angelo, Via Cintia 4, 80126 Napoli, Italy; antonella.delicato@unina.it (A.D.); vcalabro@unina.it (V.C.); 4 Dipartimento di Agraria, Sezione di Patologia Vegetale ed Entomologia, Università degli Studi di Sassari, Viale Italia 39, 07100 Sassari, Italy; lmaddau@uniss.it

**Keywords:** fungal secondary metabolites, toxins, biological activity, biofilm, antibiotic-resistance, sphaeropsidin A, *epi*-epoformin

## Abstract

Many pathogens involved in human infection have rapidly increased their antibiotic resistance, reducing the effectiveness of therapies in recent decades. Most of them can form biofilms and effective drugs are not available to treat these formations. Natural products could represent an efficient solution in discovering and developing new drugs to overcome antimicrobial resistance and treat biofilm-related infections. In this study, 20 secondary metabolites produced by pathogenic fungi of forest plants and belonging to diverse classes of naturally occurring compounds were evaluated for the first time against clinical isolates of antibiotic-resistant Gram-negative and Gram-positive bacteria. *epi*-Epoformin, sphaeropsidone, and sphaeropsidin A showed antimicrobial activity on all test strains. In particular, sphaeropsidin A was effective at low concentrations with Minimum Inhibitory Concentration (MIC) values ranging from 6.25 μg/mL to 12.5 μg/mL against all reference and clinical test strains. Furthermore, sphaeropsidin A at sub-inhibitory concentrations decreased methicillin-resistant *S. aureus* (MRSA) and *P. aeruginosa* biofilm formation, as quantified by crystal violet staining. Interestingly, mixtures of sphaeropsidin A and *epi*-epoformin have shown antimicrobial synergistic effects with a concomitant reduction of cytotoxicity against human immortalized keratinocytes. Our data show that sphaeropsidin A and *epi*-epoformin possess promising antimicrobial properties.

## 1. Introduction

In the last century, the use of antibiotics has played a fundamental role in extending the average human life. They allow us to treat trivial or serious infections and to carry out complex medical and surgical procedures that would otherwise result in very high mortality. However, the excessive or inappropriate use of antibiotics, in human medicine but also zootechnics and agriculture, results in bacterial strains possessing increased antibiotic resistance and isolation frequency.

The ability to transfer resistance determinants horizontally makes the bacterium frequently acquire resistance to multiple classes of drugs and consequently infection treatment becomes complicated. Indeed, multidrug-resistant bacteria are currently considered a global health challenge.

On the other hand, the development of new drugs is very slow due to lack of investment. The alarming epidemiological data prompted the WHO to predict that after 2050 deaths from previously treatable infections will be 10 million per year if no action is taken [1]. Increased antibiotic resistance has reduced the effectiveness of therapies by promoting the persistence of infections. The chronicity of many infections is also promoted by the ability of multiple pathogens to form biofilms on biotic or abiotic surfaces [2,3,4]. A biofilm is a mono- or polymicrobial community of cells embedded in an exopolysaccharide matrix. Within the biofilm, bacteria may be less susceptible to both the effectors of the immune response and antimicrobial drugs, so persistence of the infection is favored. The WHO has estimated that about 80% of chronic infections are related to the formation of biofilms. Many of the drugs currently available hardly penetrate the biofilm and the bacteria in the biofilm are 10–1000 times more resistant than the planktonic counterpart [5].

In this scenario, alternative strategies to conventional antimicrobial therapies are necessary and urgent. Among them, photodynamic therapy (PDT), first considered for the treatment of specific types of cancer [6], has been paid increasing attention as an innovative treatment to eradicate localized infections, supported by antibiotic-resistant biofilm-producing bacteria. [7,8]. Various studies report strategies to prevent biofilm formation on medical devices such as catheters, sutures, stents and bone cement [9,10,11,12]. Several researchers are involved in the design and evaluation of specific molecules with potential therapeutic use in chronic and biofilm-related infections [13,14]. In particular, antimicrobial peptides (AMPs), a small bioactive protein consisting of 12–50 amino acids, seem to have emerged as promising active agents against bacteria, viruses, fungi, and also as potential chemotherapeutic agents [15,16,17,18].

Plants and microorganisms have always been an invaluable source of secondary metabolites (SM) that could represent an efficient solution to this problem. SM are usually low molecular weight organic compounds produced by various organisms through the action of different enzymes. These specialized metabolites are often not essential for the growth, development, or reproduction of those organisms producing them, but they could be very important for functions such as protection, competition, and species interactions. Most of the SM isolated from microorganisms and plants have been shown to possess a broad spectrum of biological activities including antimicrobial properties. Furthermore, SM belong to diverse structural classes of naturally occurring compounds and have different mechanisms of action, a potential for the development of new drugs to overcome antimicrobial resistance and to treat biofilm-related infections [19,20,21,22,23].

Among the terrestrial ecosystems, forests represent an enormous reservoir of pathogenic and endophytic fungi, which have been studied for several years to evaluate their ability to biosynthesize phytotoxic metabolites. However, several SM produced by these organisms also possess other biological activities (including antibacterial properties) and most of them have shown potential applications in other fields such as medicine and agriculture [24]. 

Thus, in our continuing effort to find new natural antibacterial metabolites, 20 secondary metabolites produced by pathogenic fungi of forest plants and belonging to different classes of naturally occurring compounds, such as butenolides, cyclohexen oxides, diterpenes, isobenzofuranones, isocoumarins, macrolides, etc., were evaluated for the first time against reference and clinical strains of antibiotic-resistant staphylococci and *P. aeruginosa*.

## 2. Results and Discussion

**Figure 1:**
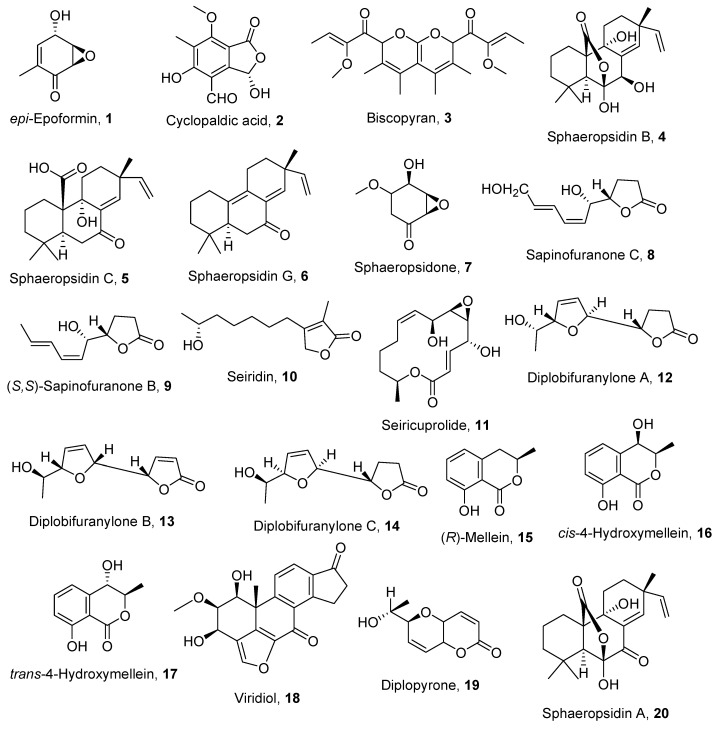
The structures of compounds **1–20**.

The secondary metabolites assayed in this study (**1**–**20**, Figure 1) were isolated as phytotoxins produced by different fungal genera responsible for forest plant diseases such as *Diplodia, Seiridium, Biscogniauxia, Sardiniella* and *Hymenoscyphus* (Table 1) and are potentially involved in plant pathogenesis [25,26,27,28,29,30,31,32,33,34,35,36]. 

However, most of these compounds also showed other interesting biological activities as reported in detail in a recent review [24]. In particular, some of them have already been reported for their antifungal activity, such as cyclopaldic acid, *epi*-epoformin, sphaerosidins A–C, sphaeropsidone and (*R*)-mellein [24,25,26,30,37,38]. In addition, **1** and **2** were able to inhibit the development of two major rust fungi in agrarian crops *P. triticina* and *U. pisi* [39,40]. Compounds **2**, **10** and **20** had larvicidal and biting deterrent activity against *Aedes aegypti* (Diptera: Culicidae), the arboviruses vector responsible for dengue fever [41]. Compound **7** induced haustorium development in radicles of the parasitic weeds *Striga* and *Orobanche* [42]. Compound **20** exhibited in vitro antibacterial activity towards *Xanthomonas oryzae* pv. *oryzae*, the causal agent of rice bacterial blight [43] and showed promising anticancer activity against drug-resistant melanoma cells [44,45]. Given that the absolute configuration (AC) is strictly linked to biological activity [46,47], the AC of **12**–**14**, **19** and **20** was determined using different methods [48,49,50].

Compounds **1**–**20** were tested at a single concentration of 100 μg/mL against reference and clinical strains of antibiotic-resistant Gram positive and Gram negative bacteria. *epi*-Epoformin (**1**), sphaeropsidone (**7**), and sphaeropsidin A (**20**) showed antimicrobial activity on all test strains; the growth inhibition rates were higher than 90% for Gram-positive bacteria and ranged from 50 to 100% for Gram-negative bacteria (Table 2). Dimethylsulphoxide (DMSO), used to dissolve the tested compounds, was simultaneously assayed at increasing concentrations (ranging from 0.1% to the maximum concentration used of 1%) to evaluate a possible effect on bacterial growth. The results showed no inhibition of the test strains in the presence of any of the DMSO concentrations used (data not shown). Therefore, the Minimum Inhibitory Concentration (MIC) and Minimum Bactericidal Concentration (MBC) values of substances **1**, **7**, **20** were determined (Table 3).

Sphaeropsidin A (**20**) was effective at low concentrations with MIC values ranging from 6.25 μg/mL to 12.5 μg/mL against all reference and clinical strains of both Gram-negative and Gram-positive bacteria. This result appears to be relevant because multi-drug resistant clinical strains were used as test strains. MBCs of compound **20** ranged from 25 to 100 μg/mL against Gram-positive bacteria, while MBC values were higher than 200 μg/mL for *P. aeruginosa* strains. MIC values of compound **1** were 100 μg/mL against all tested Gram-positive bacteria, with MBCs of 100 μg/mL, suggesting a bactericidal action. MIC values of 50 μg/mL were obtained for compound **1** against *P. aeruginosa* strains, while MBC values were higher than 200 μg/mL. For compound **7**, a MIC at concentrations below 100 μg/mL was not found for any of the test strains; the MBC values were all above 200 μg/mL.

In order to obtain increased antimicrobial activity using lower concentrations of the more active compounds, we evaluated the potential synergistic effect of sphaeropsidin A (**20**) in combination with *epi*-epoformin (**1**) or sphaeropsidone (**7**).

**Figure 2:**
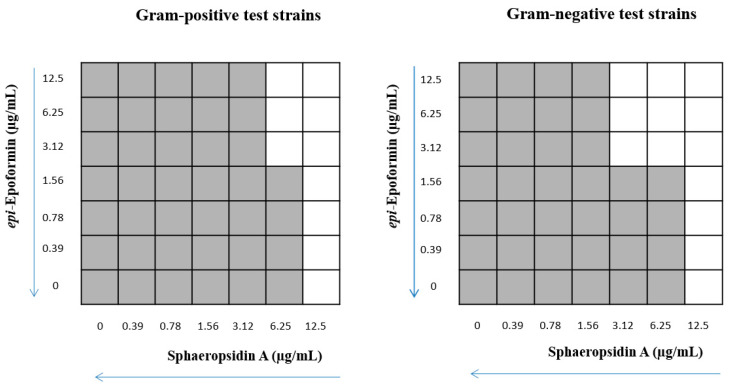
Multi-wells model of checkerboard assay to test the interaction between *epi*-epoformin and sphaeropsidin A. Turbidity was reported in grey and no bacterial growth in white.

Compound **7** showed no synergistic effect in combination with sphaeropsidin A (Fractional Inhibitory Concentration (FIC) index = 2, data not shown). For the combination of *epi*-epoformin with sphaeropsidin A, we obtained the highest synergistic interaction against Gram-positive bacteria at a concentration of 6.25 μg/mL sphaeropsidin A (1/2 MIC) and 3.12 μg/mL *epi*-epoformin (1/32 MIC). The highest synergistic interaction against Gram-negative bacteria was obtained at 3.12 μg/mL *epi*-epoformin (1/16 MIC) and 3.12 μg/mL sphaeropsidin A (1/4 MIC) (Figure 2).

The FIC index showed a synergistic effect of *epi*-epoformin and sphaeropsidin A against Gram-negative strains (FIC index < 0.5), and an additive effect against Gram-positive strains (0.5 ≤ FIC index ≤ 1.0).

**Figure 3:**
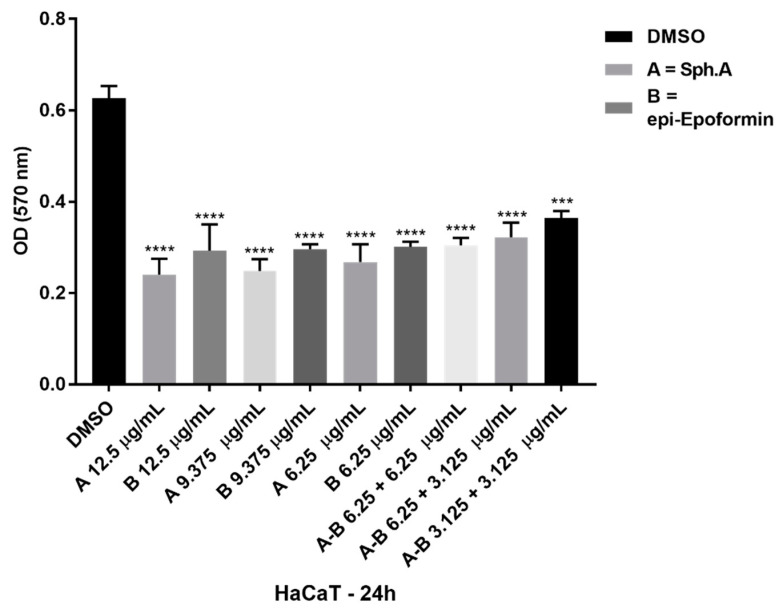
MTT viability test. Hacat cells were incubated with the indicated amount of spheropsidin A (compound A) and *epi*-epoformin (compound B) alone or in combination (AB) for 24 h. The MTT viability test was performed as described in Material. The values were the mean’s three values for each experimental point of two biological replicates. Each pair of means were compared using a Tukey’s multiple comparisons test *p*-value < 0.05, *** *p* < 0.001; **** *p* < 0.0001)

The cytotoxic activity of metabolites **1**, **7**, and **20** was evaluated on human spontaneously immortalized HaCat keratinocytes. HaCat cells were treated at a concentration ranging from 3.12 μg/mL to 100 μg/mL of metabolites **1**, **7**, and **20** for 24 h and then subjected to the 3-(4,5-dimethylthiazol-2-yl)-2,5-diphenyl tetrazolium bromide (MTT) assay. At a concentration of 100 μg/mL, all tested metabolites dramatically reduced HaCat cell viability. At 12.5 and 6.25 μg/mL, sphaeropsidin A reduced cell viability to 38% and 43%, respectively. However, when HaCat cells were treated with a mixture of 3.12 μg/mL of sphaeropsidin A and 3.12 μg/mL of *epi*-epoformin, cell viability was around 60%. This combination was substantially less cytotoxic than 6.25 μg/mL of each compound alone (Figure 3). This result is encouraging given that the combination of these two metabolites, at 3.12 μg/mL each, was shown to synergize against Gram-negative strains. A mixture of 6.25 μg/mL of sphaeropsidin A and 3.12 μg/mL of *epi*-epoformin showed an additive effect against Gram-positive bacteria. This combination reduced HaCat cell viability to 54% and was slightly less cytotoxic than 9.37 μg/mL of each compound alone (Figure 3).

**Figure 4:**
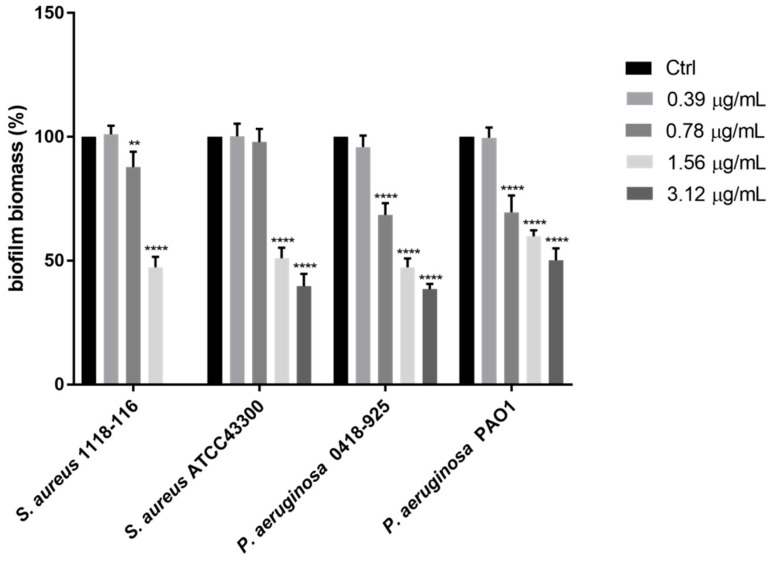
In vitro biofilm formation of test strains following overnight treatment with compound **20** at serial dilutions of sub-MIC concentrations. Biofilm formation was determined by cristal violet assay. Values are presented as mean percentage ± SD. ** *p*-value = 0.009, **** *p*-value < 0.001.

The persistence of infections is also frequently favored by the ability of bacteria to grow in the form of biofilm, within which the microorganism is protected from the host’s response as well as from many antimicrobial agents. Sphaeropsidin A (**20**) was also tested for its potential ability to inhibit biofilm formation, starting from sub-MIC concentrations that had shown no influence on the planktonic growth of the test strains (data not shown). In comparison with the untreated control, compound **20** was able to reduce the adhesion of *P. aeruginosa* clinical and reference strains by 62% and 50%, respectively, at a concentration of 3.12 μg/mL, corresponding to 1/4 MIC. The biofilm formation of MRSA clinical and reference strains was inhibited by 53% and 60% at the concentration of 1.56 μg/mL and 3. 12 μg/mL, respectively, corresponding to 1/4 MIC of compound **20** (Figure 4). 

The formation of biofilm by *S. haemolyticus* strains did not appear to be inhibited by sphaeropsidin A. This latter, tested in synergy with *epi*-epoformin in combinations lower than the synergistic concentrations inhibiting planktonic growth, did not influence the anti-biofilm effect showed by sphaeropsidin A alone (data not shown).

*S. aureus*, as commensal of the skin, and *P. aeruginosa*, as an environmental saprophyte, are the most frequent opportunistic pathogens causing infections of surgical and traumatic wounds and burns [51,52,53,54]. The isolation of antibiotic resistant strains is continuously increasing [55,56,57]. Moreover, their attachment to host tissues, as well as to medical implants and the production of biofilm, play an important role in the persistence of these infections [58,59]. The establishment of a mature biofilm, which is significantly less sensitive to antimicrobial agents than genetically identical non-adherent planktonic cells, considerably delays the healing process [4,60,61]. A biofilm-focused therapeutic approach, that reduces the ability of these pathogens to form biofilms, would decrease the antibiotic recalcitrance of these infections, thus allowing treatment with the antibiotics in use, and faster and more effective healing. Therefore, our data seems to be interesting since sphaeropsidin A appears capable of reducing biofilm formation by all the test strains. To our knowledge, this is the first report on the anti-biofilm activity of sphaeropsidin A. This compound was able to inhibit the biofilm of clinical MRSA at a concentration of 1.56 μg/mL; this result encourages further studies on a greater number of clinical strains and on other bacterial species. In addition, *epi*-epoformin and sphaeropsidin A synergized against Gram-negative and showed an additive effect against Gram-positive bacteria. However, the clinical value of a drug is strictly dependent on the evaluation of its cytotoxicity for the host cells, but sphaeropsidin A and *epi*-epoformin did not show sufficient selectivity between bacteria and eukaryotic cells. Nevertheless, in our opinion, the screening performed represents a promising basis to identify scaffolds with antimicrobial potential. *epi*-Epoformin, spheropsidone and sphaeropsidin A (**1**, **7** and **20**) contain structural features known to be responsible for such activity in naturally occurring compounds [62]. The functionalities are the epoxy group in **1** and **7** and the α, β-unsaturated ketone group in **1** and **20**, which could react with a nucleophilic group of the receptor (such as -NH_2_ or -SH, etc.). In fact, the epoxide, through a bimolecular nucleophilic substitution (SN_2_), and the α, β-unsaturated carbonyl group, through a Michael addition, could yield conjugates with a stable covalent bond, which could also be shown using spectroscopic techniques such as MS-TOF [63]. The knowledge of the action mechanism of these compounds could suggest chemical modifications of their structure to synthesize derivatives with acceptable biocompatibility and improved antimicrobial properties against multi-resistant and biofilm-producing bacteria.

## 3. Conclusions

Our results represent preliminary data on the antimicrobial activity of fungal secondary metabolites evaluated for the first time against clinical isolates of *P. aeruginosa* and *S. aureus*, considered as common opportunistic pathogens inducing severe human infections. Sphaeropsidin A activity appears noteworthy for its ability to inhibit biofilm formation, preventing a growth mode that results in particular resistance to antibiotic treatment. The results obtained are preliminary to further experiments aimed at developing biocompatible formulations of *epi*-epoformin and sphaeropsidin A, suitable for wound treatment to prevent the development of serious infections.

## 4. Materials and Methods 

### 4.1. General Experimental Procedures

The secondary metabolites used in this study (**1**–**20**, Figure 1) have been isolated from pathogenic fungifollowing procedures previously reported and listed in Table 1. All the data regarding their source, their chemical family and literature are reported in Table 1. The purity of each compound was >98%, as ascertained by TLC, ESI-MS and NMR using well-established methods. Analytical and preparative thin-layer chromatography (TLC) was performed on silica gel (Kieselgel 60, F_254_, 0.25 and 0.5 mm respectively) plates (Merck, Darmstadt, Germany); the spots were visualized by exposure to UV light or by spraying with 10% H_2_SO_4_ in CH_3_OH and then 5% phosphomolybdic acid in EtOH, followed by heating at 110 °C for 10 min. ESI-MS spectra were recorded on Agilent Technologies 6120 quadrupole LC/MS instrument (Agilent instruments, Milan, Italy); ^1^H NMR spectra were recorded at 400 MHz, on Bruker spectrometer (Bruker BioSpin GmbH., Karlsruhe, Germany), using the same solvent as an internal standard.

### 4.2. Microbial Strains and Culture Conditions

Bacterial strains used in this study were methicillin-resistant *Staphylococcus aureus* ATCC 43300, *Staphylococcus haemolyticus* ATCC 29970 and *Pseudomonas aeruginosa* PAO1 as reference strains, and three multi-drug resistant clinical isolates: methicillin-resistant *S. aureus* (MRSA) 1118-116, methicillin and vancomycin-resistant *S. haemolythicus* (VRSH) 1219-118, and extended-spectrum beta-lactamase (ESBL) producing *P. aeruginosa* 0418-925. The strains were obtained from a collection previously established at the Department of Molecular Medicine and Medical Biotechnologies (University of Naples Federico II). No ethical approval was required for the study because there was no access to patients’ data. All strains were stored as 15% (v/v) glycerol stocks at −80 °C. Before each experiment, cells were sub-cultured from the stocks onto TSA plates at 37 °C for 24 h. Identification was performed by biochemical characterization using the Vitek2 (Biomerieux, Mercy-l’Etoile, France) and Phoenix (Becton Dickinson, Sparks, MD, USA) systems and confirmed by MS MALDI-TOF (Bruker Daltonics, Bremen, Germany). Susceptibility to antibiotics was assessed using automatic (Vitek2; Phoenix) and Kirby Bauer disk diffusion (Thermo Fisher Scientific, Basingstoke, UK) antibiotic sensitivity testing.

### 4.3. Antimicrobial Assays

The initial screening of 20 fungal metabolites was performed by standard broth micro-dilution assay in 96-wells polystyrene plates using Mueller-Hinton Broth 2 (MHB2) to test a single high concentration against all test strains. Briefly, 2 × stock solutions of all compounds were made by dissolving them in 2% DMSO. For each strain, the cell suspension was prepared at 0.5 McFarland standard (corresponding to approximately 10^8^ CFU/mL) and subsequently adjusted to approximately 5 × 10^6^ CFU/mL^−1^. One hundred µL aliquots (5 × 10^5^ CFU) of these bacterial suspensions were treated with 100 µL of 100 µg/mL solution of the compound under investigation; wells with only MHB2 were used as negative control and wells with no compounds as positive growth control. The effect of serial dilutions of DMSO starting from 1% on the growth of test strains was separately tested. Plates were incubated at 37 °C for 19 h under shaking (300 rpm). Then the medium turbidity was measured by a microtiter plate reader at 595 nm (Bio-rad Laboratories s.r.l.). Antimicrobial activity was expressed as a percentage of microbial growth inhibition. Each compound was tested in triplicate and each experiment was performed twice. Minimal inhibitory concentration (MIC) and Minimal Bactericidal Concentration (MBC) of selected compounds were determined by the broth micro-dilution assay. The starting inoculum was 5 × 10^6^ CFU mL^−1^, and the concentrations of the metabolites ranged from 200 to 0.78 µg/mL (twofold dilutions). As positive controls, conventional antimicrobials, selected depending on antibiotic-susceptibility profiles of the test strains, were included: amikacin (ranged from 32 to 2 µg/mL) was used for Gram-negative strains and teicoplanin (ranged from 0.5 to 4 µg/mL) for Gram-positive strains. Medium turbidity was measured by a microtiter plate reader at 595 nm. The MIC was defined as the lowest concentration of compound that caused ≥90% inhibition of bacterial growth. The MBCs were determined by transferring 200 µL of each sample, previously treated with compound concentrations equal to or higher than the MIC, onto TSA plates and incubating the plates at 37 °C for 24/48 h. The lowest compound concentration that yields no microbial growth on agar plates will be defined as the MBC. Each compound was tested in triplicate; each experiment was performed twice.

### 4.4. Synergy Assays

The interactions between selected metabolites were evaluated by the checkerboard method in 96-well microtiter plates [64]. The compounds to be tested in combination were serially diluted, one along with the x-axis and the other along with the y-axis. The final compounds’ concentrations (after two-fold dilutions) varied from 0.19 µg/mL up to the 12.5 µg/mL for each one. The checkerboard plates were inoculated with test strains at a concentration of 5 × 10^6^ CFU/mL and incubated at 37 °C for 19 h, then the microbial growth was visually assessed, and the turbidity measured by the microplate reader at 595 nm. To evaluate the effect of the combination treatment, the fractional inhibitory concentration (FIC) index for each combination was calculated as follows: FIC index = FIC of compound A + FIC of compound B, where FIC of compound A (or compound B) will be defined as the ratio of MIC of compound A (or compound B) in combination and MIC of compound A (or of compound B) alone. The FIC index values are interpreted as follows: ≤0.5, synergistic; >0.5 to ≤1.0, additive; >1.0 to ≤2.0, indifferent; and >2.0, antagonistic effects [64].

### 4.5. Biofilm Formation Inhibition Assay

The total biomass of the biofilm was analyzed using the Crystal Violet (CV) staining method in flat-bottomed 96-well microplates as described by Stepanović et al. [65]. For each strain, a cell suspension in MHB2 supplemented with 10% (w/v) glucose was prepared for turbidity of 0.5 McFarland. This suspension was further diluted at 1:100 and 100 µL of the suspension (1 × 10^6^ CFU/mL) were incubated with 100 µL of MHB2 containing the selected compound at serial dilutions of sub-MIC concentrations. The negative control was prepared by inoculating 200 µL of a microbial suspension inactivated by boiling. The positive controls were compound-free wells. To assess biofilm formation, the culture broth was gently aspirated, and each well was washed twice with PBS to remove exclusively non-adherent cells and dried at 60 °C for 45 min. The biofilm was stained by incubation for 30 min with 100 µL of a 0.1% (w/v) crystal violet solution. Any excess of crystal violet was removed by washing with PBS before adding 200 µL of absolute ethanol to release the dye from the biofilm. The absorbance was measured at 595 nm by a microplate reader and was related to the amount of biofilm produced. The percentage of biofilm mass reduction was calculated using the formula: [(Ac-At)/Ac] × 100, where Ac is the OD595 for control wells and At is the OD595 in the presence of the tested compound.

### 4.6. Cytotoxicity Test

HaCaT (human spontaneously immortalized keratinocytes from adult skin) were purchased from Cell Line Service (CLS, Hattersheim am Main, Germany). Cells were cultured in DMEM High Glucose (Gibco BRL Thermo Fisher, Milan, Italy) supplemented with 10% Fetal Bovine Serum (Gibco BRL, Thermo Fisher, Milan, Italy), 1% L-Glutamine (Gibco BRL) and 1% Pen-Strep solution (Gibco BRL) in a humidified incubator at 37 °C and 5% CO_2_. Cells were routinely checked for mycoplasma contamination, using a mycoplasma detection kit (ABM, Vancouver, Canada). Cytotoxicity was determined by the MTT 3-(4,5-dimethylthiazol-2-yl)-2,5-diphenyl tetrazolium bromide assay (Sigma-Aldrich, St Louis, MO, USA). HaCaT were seeded in 24-well plates at 3.0 × 10^4^ per well and treated with sphaeropsidin A, sphaeropsidone, and *epi*-epoformin at concentrations between 3.125 µg/mL and 100 µg/mL for 24 h. The assay was performed according to the manufacturer’s instructions. The optical absorbance was determined at 570 nm and 630 nm using an iMark microplate reader (Bio-Rad, Milan, Italy). Each value shown in the plot is mean ± SD of triplicate determinations. Asterisks represent significant results (*** *p >* 0.001; **** *p* < 0.0001).

### 4.7. Statistical Analysis

Statistical analyses were carried out using the GraphPad Prism 8 software (San Diego, CA). Data were represented as the mean ± standard deviation and analyzed for statistical significance using ordinary one-way or two-way analysis of variance (ANOVA) and multiple comparisons. For all tests, *p* < 0.005 was considered to indicate a statistically significant difference.

## Figures and Tables

**Table 1:** Fungal metabolites used in this study.

Number	Name	Chemical Family	Fungal Source	Ref.
**1**	*epi*-Epoformin	Cyclohexene oxide	*Diplodia quercivora*	[25]
**2**	Cyclopaldic acid	Isobenzofuranone	*Seiridium cupressi*	[26]
**3**	Biscopyran	Pyranopyran	*Biscogniauxia mediterranea*	[27]
**4**	Sphaeropsidin B	Diterpenoid	*Diplodia cupressi*	[28]
**5**	Sphaeropsidin C	Diterpenoid	*D. cupressi*	[28]
**6**	Sphaeropsidin G	Diterpenoid	*Diplodia corticola*	[29]
**7**	Sphaeropsidone	Cyclohexene oxide	*D. cupressi*	[30]
**8**	Sapinofuranone C	Furanone	*D. corticola*	[31]
**9**	(*S,S*)-Sapinofuranone B	Furanone	*D. corticola*	[31]
**10**	Seiridin	Butenolide	*S. cupressi*	[32]
**11**	Seiricuprolide	Macrolide	*S. cupressi*	[33]
**12**	Diplobifuranylone A	Furanone	*D. corticola*	[31]
**13**	Diplobifuranylone B	Furanone	*D. corticola*	[31]
**14**	Diplobifuranylone C	Furanone	*D. corticola*	[31]
**15**	(*R*)-Mellein	3,4-Dihydroisocoumarin	*Sardiniella urbana*	[34]
**16**	*cis*-4-Hydroxymellein	3,4-Dihydroisocoumarin	*S. urbana*	[34]
**17**	*trans*-4-Hydroxymellein	3,4-Dihydroisocoumarin	*S. urbana*	[34]
**18**	Viridiol	Furanosteroid	*Hymenoscyphus fraxineus*	[35]
**19**	Diplopyrone	Pyranopyrone	*D. corticola*	[36]
**20**	Sphaeropsidin A	Diterpenoid	*D. corticola*	[31]

**Table 2:** Antibacterial activity, expressed as the percentage of growth inhibition, of **1**–**20** at the 100 μg/mL concentration against Gram-positive and Gram-negative test strains ^1,2^.

Compound	Bacterial Strain					
	*S. aureus* ATCC 43300	*MRSA*1118-116	*S. haemolyticus* ATCC 29970	*S. haemolyticus* VR 1219-118	*P. aeruginosa* PAO1	*P. aeruginosa* 0418-925
**1**	≥90	≥90	≥90	≥90	≥90	≥90
**2**	≥90	≥90	≥90	≥90	-	≥90
**3**	-	-	-	-	50	60
**4**	≥90	≥90	≥90	≥90	-	60
**5**	-	-	-	-	60	60
**6**	≥90	≥90	≥90	≥90	-	-
**7**	≥90	≥90	≥90	≥90	50	≥90
**8**	-	-	-	-	60	60
**9**	-	-	-	-	60	60
**10**	-	-	-	-	50	60
**11**	-	-	-	-	60	50
**12**	-	-	-	-	50	50
**13**	-	-	-	-	50	60
**14**	-	-	-	-	50	60
**15**	-	-	-	-	60	60
**16**	-	-	-	-	60	70
**17**	-	-	-	-	60	60
**18**	≥90	≥90	≥90	≥90	-	-
**19**	-	-	-	-	-	-
**20**	≥90	≥90	≥90	≥90	≥90	≥90
**AK**	nt	nt	nt	nt	>90	>90
**TE**	>90	>90	>90	>90	nt	nt

^1^ For inhibition values below 50%, no data have been reported (-). AK = Amikacin; TE = Teicoplanin; nt = not tested. ^2^ Amikacin (32 μg/mL) and teicoplanin (4 μg/mL) were used as positive controls.

**Table 3:** MIC (μg/mL) and MBC (μg/mL) of compounds **1**, **7**, **20** against Gram-positive and Gram-negative test strains ^1^.

Bacterial Strain	Compound 1		Compound 7		Compound 20		Amikacin		Teicoplanin	
	MIC	MBC	MIC	MBC	MIC	MBC	MIC	MBC	MIC	MBC
*S. aureus* ATCC 43300	100	100	100	>200	12.5	100	nt	nt	1	4
*MRSA* 1118-116	100	100	100	>200	6.25	25	nt	nt	0.5	4
*S. haemolyticus* ATCC 29970	100	100	100	>200	12.5	50	nt	nt	2	>4
*S. haemolyticus* VR 1219-118	100	100	100	>200	12.5	50	nt	nt	2	>4
*P. aeruginosa* PAO1	50	>200	>100	>200	12.5	>200	4	32	nt	nt
*P. aeruginosa* 0418-925	50	>200	100	>200	12.5	>200	16	>32	nt	nt

^1^ Amikacin and teicoplanin were used as positive controls; nt = not tested.
